# Coefficient of R‐R interval variations under deep breathing load in patients with wild‐type transthyretin amyloid cardiomyopathy: A case‐control study

**DOI:** 10.1002/hsr2.938

**Published:** 2022-11-30

**Authors:** Yasuhiro Nagayoshi, Hiroaki Kawano, Taiki Nishihara, Kei Morikawa, Haruka Nagano, Shinsuke Hanatani, Naritsugu Sakaino, Kenichi Tsujita

**Affiliations:** ^1^ Department of Cardiology Amakusa Medical Center Amakusa Japan; ^2^ Department of Cardiovascular Medicine, Graduate School of Medical Sciences Kumamoto University Kumamoto Japan

**Keywords:** autonomic dysfunction, cardiac amyloidosis, heart rate variability

## Abstract

**Background and Aims:**

An autonomic nervous disorder is an important characteristic of cardiac amyloidosis; however, the prevalence of autonomic dysfunction in wild‐type transthyretin amyloidosis (ATTR_wt_) has not been established. Analysis of the R‐R interval coefficient of variation (CVR‐R) is a noninvasive method to measure parasympathetic activity. We aimed to assess autonomic dysfunction of ATTR_wt_ and determine the utility of CVR‐R for the detection of ATTR_wt_ in other cardiac diseases.

**Methods:**

This is a single‐center, retrospective, case‐control study. Fifty patients with heart failure (HF) were studied. The etiologies of HF were as follows: ATTR_wt_, *n* = 10; previous myocardial infarction (MI), *n* = 20; and left ventricular hypertrophy (LVH) due to other disease processes (e.g., aortic stenosis), *n* = 20. We measured the CVR‐R at rest (CVR‐R_rest_), CVR‐R with deep breaths (CVR‐R_breath_), and the change rate (CVR‐R_diff rate)_. The relative change formula is as follows: CVR‐R_diff rate_ = (CVR‐R_breath_ − CVR‐R_rest_)/CVR‐R_rest_ × 100 (%).

**Results:**

There was no difference in the CVR‐R_rest_ levels among the three groups. The CVR‐R_diff rate_ levels in the ATTR_wt_ group were significantly lower (ATTR_wt_: −8.77 [−43.8 to 10.9]; LVH: 67.4 [38.7 to 89.4]; MI: 83.7 [60.4 to 142.9]). Based on the receiver operative characteristic curve analysis to identify ATTR_wt_ in HF, the best cut‐off value for the CVR‐R_diff rate_ was 19.7 (area under the curve: 0.848).

**Conclusion:**

Our data suggested autonomic dysfunction in patients with ATTR_wt_. Measurement of the CVR‐R in HF patients may be a convenient support tool for the detection of ATTR_wt_.

## 1 INTRODUCTION

Cardiac amyloidosis (CA) is a progressive disease caused by myocardial deposition of amyloid fibrils.^1^ There are many different types of amyloidosis, but CA is mainly divided into the following three types: immunoglobulin light chain amyloidosis (AL amyloidosis); hereditary transthyretin amyloidosis (ATTR_v_); and wild‐type transthyretin amyloidosis (ATTR_wt_). The major findings of CA are left ventricular hypertrophy (LVH) and a cardiac conduction disorder. An autonomic nervous system disorder is one of the important features.^2^ Heart rate variability (HRV) is a widely used parameter to assess autonomic function.^3^ Indeed, HRV has been reported to be a prognostic indicator for CA.^4^, ^5^, ^6^ Autonomic dysfunction is frequently observed in ATTR_v_ and AL amyloidosis. Reduction of the standard deviation of beat‐to‐beat or NN intervals (SDNN) ≤ 50 ms is a strong predictor of 1‐year mortality in patients with AL amyloidosis^4^; however, the prevalence of autonomic dysfunction and the usefulness of HRV in patients with ATTR_wt_ has not been established. The coefficient of variation in R‐R intervals (CVR‐R) is a measure of HRV that mainly reflects the parasympathetic nervous system. The aims of this study were: (1) to assess autonomic dysfunction of ATTR_wt_ using the CVR‐R and (2) to determine the utility of the CVR‐R for detecting ATTR_wt_ in patients with other cardiac conditions of LVH or myocardial infarction.

## 2 METHODS

This is a single‐center, retrospective, case‐control study. First, we enrolled the consecutive patients with ATTR_wt_ (70–90 years of age) attending to our hospital between April 2019 and March 2021. ATTR_wt_ was diagnosed by endomyocardial biopsy and/or ^99m^Tc‐PYP scintigraphy. Amyloid deposition was identified by Congo red staining in combination with polarization microscopy and classified by immunohistochemistry. The presence of global transmural or subendocardial late gadolinium enhancement on cardiac magnetic resonance added support to the diagnosis of ATTR_wt_. As a comparison with ATTR_wt_ group, we subsequently enrolled the consecutive HF patients (70–90 years of age) with LVH or previous myocardial infarction (MI), who underwent CVR‐R test during the same period. These patients were assessed using New York Heart Association (NYHA) classification. The LVH group included patients with hypertrophic cardiomyopathy (HCM), aortic stenosis, and hypertensive heart disease. The clinical diagnosis of HCM was made based on echocardiographic demonstration of LVH with an end‐diastolic wall thickness ≥15 mm in more than 1 segment in the absence of any disease which causes LVH. A history of MI was confirmed with the patient's medical record. The HF subtypes were defined according to European Society of Cardiology guidelines: HF with preserved ejection fraction as LVEF ≥ 50%, HF with midrange ejection fraction as 40%–49%, and HF with reduced ejection fraction as LVEF < 40%.^7^

Patients with arrhythmias (e.g., atrial fibrillation, ventricular premature contractions, II–III atrioventricular block, paced rhythm, and a history of catheter ablation of cardiac arrhythmias) were excluded. We also excluded patients with NYHA classification IV, AL amyloidosis, AA amyloidosis, ATTR_v_, and a history of a MI that occurred 1 year before enrollment. In the absence of a genetic diagnosis, ATTR_v_ was ruled out based on the family medical history. A small number of registrations was expected because of the rarity of the disease, strict eligibility criteria, and the single‐center study. Study sample size was referenced from previous studies.^5^, ^6^

Blood samples were collected from patients who were clinically stable. Serum cardiac troponin I and plasma brain natriuretic peptide (BNP) levels were measured in all patients. The chronic kidney disease (CKD) stage was based on the estimated glomerular filtration rate (e‐GFR), which was calculated using the Modification of Diet in Renal Disease (MDRD) equation. Anemia was defined as a hemoglobin level < 13.0 g/dl in men and <11.5 g/dl in women. Hypertension, dyslipidemia and diabetes mellitus were diagnosed according to their guidelines, respectively.^8^, ^9^, ^10^

Echocardiographic parameters included chamber size, wall thickness, left ventricular ejection fraction (LVEF), and left ventricular mass. LVEF was calculated using Simpson's method. The peak early and late diastolic velocity of left ventricular inflow (E and A waves, respectively) and the deceleration time of the E wave were determined. The peak diastolic velocity on the septal and lateral corner of the mitral annules was measured in the apical four‐chamber view and the E/e’ ratio was calculated. The left ventricular mass index (LVMI) was calculated based on the following formula: {0.8 × (1.04[(LVDd + PWT + IVST)3 – (LVDd)3]) + 0.6}/body surface area, where LVDd = left ventricular end‐diastolic dimension, IVSTd = interventricular septal thickness at end‐diastole, and PWTd = posterior wall thickness at end‐diastole. The relative apical sparing and right ventricular (RV) free wall thickness were evaluated.

After at least 5 min at rest, a standard 12‐lead ECG was recorded in the spine position (VS‐3000E; Fukuda Denshi), and the R‐R interval was analyzed for 1 min. Drugs with anticholinergic effects were discontinued before testing. Following measurement of the R‐R interval with normal breathing, the R‐R interval with deep breaths at a rate of six times/min was analyzed. Based on the mean R‐R interval (mRR) and the R‐R standard deviation (RR‐SD), the CVR‐R was calculated as follows: RR‐SD/mRR × 100(%). In healthy persons, CVR‐R values are higher during deep breaths than at rest. The CVR‐R values in normal control subjects were not examined in this study. According to the previous reports, the CVR‐R values in normal elderly subjects were about 2.0%–3.0%.^11^, ^12^ Deep breathing load increases the values about two times.^12^, ^13^ The CVR‐R values vary depending on the patients’ characteristics. For this reason, we examined the CVR‐R values in LVH and MI as control. Following measurement of the CVR‐R at rest (CVR‐R_rest_) and the CVR‐R with deep breathing (CVR‐R_breath_), the difference rate (CVR‐R_diff rate_) was calculated using the following formula: CVR‐R_diff rate_ = (CVR‐R_breath_ – CVR‐R_rest_)/CVR‐R_rest_ × 100 (%).

Continuous data are summarized by the mean ± SD or median (interquartile range) if the distribution was skewed. Categorical data are reported as numbers and percentages. Multiple group comparisons of continuous data were performed with one‐way analysis of variance (ANOVA) or the Kruskal–Wallis test. Categorical variables are presented as percentages and compared with a *χ*² or Fisher's exact test. After the Bonferroni correction for multiple comparisons, a *p*‐value < 0.05 was considered significant in all statistical analyses. Receiver‐operating characteristic (ROC) curve analysis was performed to identify CA in HF patients and to calculate sensitivity, specificity, area under the ROC curve (AUC), and the optimal cut‐off value. Cardiac troponin is a sensitive marker of CA.^14^ The AUC values of cardiac troponin I and CVR‐R_diff rate_ were compared using DeLong's method. All statistical analyses were carried out with the EZR on R‐commander (version 1.53; Saitama Medical Center, Jichi Medical University), which is a graphical user interface for R (The R Foundation for Statistical Computing).^15^

All procedures were performed in accordance with the Declaration of Helsinki and its amendments. The study protocol was approved by the Institutional Review Board of the Amakusa Medical Center (approval no. 20210316‐5). The requirement for informed consent was waived because of the low‐risk nature of this retrospective study. We announced this study protocol at the Amakusa Medical Center and on our website (http://www.amed.jp/mc/index.php) and gave patients the opportunity to withdraw from the study.

## 3 RESULTS

**Figure 1:**
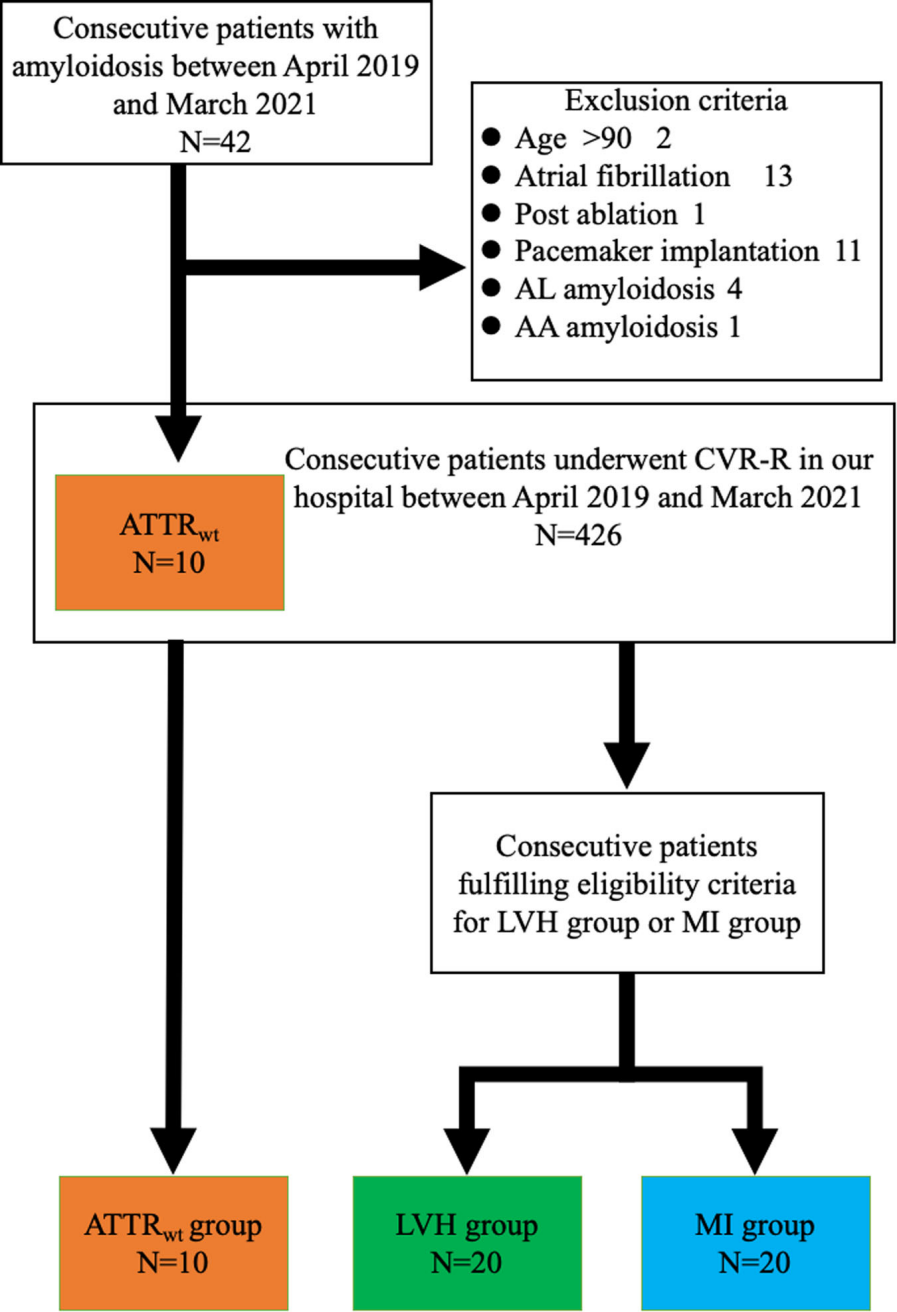
Study flow chart. Of 426 consecutive patients who underwent R‐R interval coefficient of variation (CVR‐R), consecutive 50 patients who fulfill the eligibility criteria were enrolled in this study. ATTR_wt_, wild‐type transthyretin amyloidosis; LVH, left ventricular hypertrophy; MI, previous myocardial infarction.

Forty‐two patients were diagnosed as having CA between April 2019 and March 2021, and consecutive 10 patients with ATTR_wt_ were evaluated with CVR‐R (Figure 1). Of 426 consecutive patients who underwent CVR‐R in our hospital, 50 patients who fulfill the eligibility criteria were enrolled in this study (10 ATTR_wt_, 20 LVH, and 20 MI). The clinical characteristics of the patients are shown in Table 1. There were no differences in age, gender, and co‐morbidities among the three groups, except for the prevalence of dyslipidemia. The plasma BNP and troponin I values were significantly higher in the ATTR_wt_ group. Based on the echocardiography findings, the left ventricular mass index, and mitral E/A and E/e’ were significantly higher in the ATTR_wt_ group; however, the E wave deceleration time was significantly lower in the ATTR_wt_ group. Apical sparing and right ventricular hypertrophy were also observed in the ATTR_wt_ group. The prevalence of beta blocker prescription was lower in the ATTR_wt_ group, although the difference was not significant. The use of ACE/ARB was most frequent in the MI group.

**Table 1:** Clinical characteristics of patients

	ATTR_wt_ (*n* = 10)	LVH (*n* = 20)	MI (*n* = 20)	*p*‐Value
Age	79.5 ± 5.7	78.3 ± 5.6	80.1 ± 5.7	0.609
Male (*n*, %)	9 (90%)	14 (70%)	14 (70%)	0.455
**Co‐morbidity**				
Hypertension (*n*, %)	7 (70%)	18 (90%)	18 (90%)	0.292
Diabetes mellitus (*n*, %)	2 (20%)	5 (25%)	9 (45%)	0.334
Dyslipidemia (*n*, %)	4 (40%)	10 (50%)	20 (100%)	<0.001
Chronic kidney disease (*n*, %)	6 (60%)	12 (60%)	12 (60%)	>0.99
Anemia (*n*, %)	4 (40%)	7 (35%)	13 (65%)	0.174
HbA1C (%)	5.8 (5.6–6.0)	6.1 (5.5–6.1)	6.3 (5.9–7.5)	0.104
BNP (pg/ml)	524.8 (442.1–1035.9)	172.6 (97.9–274.7)	251.4 (56.1–478.2)	0.017
Troponin I (ng/L)	0.151 (0.095–0.198)	0.035 (0.01–0.079)	0.03 (0.01–0.06)	0.004
**Revascularization therapy**				
CABG	0	1	4	0.249
PCI	0	3	15	<0.001
**Echocardiography**				
LVEF (%)	46.2 ± 12.4	63.6 ± 11.6	46.9 ± 9.0	<0.001
LVMI (g/m^2^)	181.9 ± 46.1	137.86 ± 59.5	120.1 ± 27.6	0.005
E/A	1.7 ± 0.5	0.9 ± 0.3	0.8 ± 0.3	<0.001
Deceleration time (ms)	171.3 ± 46.3	246.2 ± 81.4	233.7 ± 69.0	0.027
E/e'	21.2 ± 6.1	13.4 ± 4.6	12.5 ± 5.7	<0.001
Apical sparing	9 (90%)	0	0	<0.001
RV wall thickness	9 (90%)	0	0	<0.001
**HF subtypes**				
HFrEF (LVEF < 40%)	4 (40%)	1 (5%)	2 (10%)	
HFmrEF (LVEF 40%–49%)	3 (30%)	1 (5%)	12 (60%)	
HFpEF (LVEF ≥ 50%)	3 (30%)	18 (90%)	6 (30%)	
**Medication**				
Β‐blockers (*n*, %)	2 (20%)	10 (50%)	11 (55%)	0.204
ACE‐inhibitors/ARBs (*n*, %)	5 (50%)	13 (65%)	19 (95%)	0.015
**Coefficient of variation in R**‐**R intervals**				
CVR‐R rest	2.63 (1.85–3.02)	1.86 (1.42–2.31)	2.07 (1.28–3.08)	0.223
CVR‐R breath	2.28 (1.26–3.13)	3.42 (2.75–3.71)	3.81 (2.72–5.63)	0.008
CVR‐R difference rate (%)	−8.77 (−43.8 to 10.9)	67.4 (38.7–89.4)	83.7 (60.4–142.9)	0.002

Abbreviations: ACE, angiotensin converting enzyme; ARB, angiotensin receptor blocker; BNP, brain natriuretic peptide; CABG, coronary artery bypass graft surgery; CVR‐R, coefficient of variation of R‐R interval; HbA1C, hemoglobin A1C; HF, heart failure; HFrEF, heart failure with reduced ejection fraction; HFmrEF, heart failure with midrange ejection fraction; HFpEF, heart failure with preserved ejection fraction; IVSTd, intraventricular septal thickness at end‐diastole; LVDd, left ventricular end‐diastolic diameter; LVDs, left ventricular systolic dimension; LVEF, left ventricular ejection fraction; LVH, left ventricular hypertrophy; LVMI, left ventricular mass index; MI, previous myocardial infarction; PCI, percutaneous coronary intervention; PWTd, posterior wall thickness at end‐diastole; RV, right ventricular.

**Figure 2:**
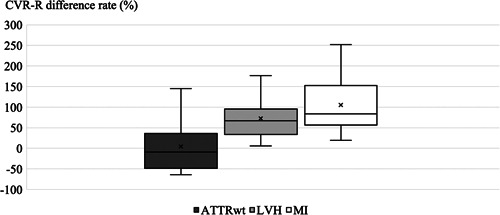
Comparison of the CVR‐R change rate under deep breathing between the ATTR_wt_, LVH, and MI groups. The CVR‐R change rate under deep breathing (CVR‐R_diff rate)_ in the ATTR_wt_ group was significantly lower than the LVH and MI groups (−8.77 [−43.8 to 10.9] *n* = 10, 67.4 [38.7 to 89.4] *n* = 20 and 83.7 [60.4 to 142.9] *n* = 20, *p*‐value < 0.01). ATTR_wt_, wild‐type transthyretin amyloidosis; CVR‐R, coefficient of variation of R‐R interval; LVH, left ventricular hypertrophy; MI, previous myocardial infarction.

There was no difference in the CVR‐R_rest_ levels among the three groups. In the LVH and MI groups, the CVR‐R_breath_ was higher than the CVR‐R_rest_; this change was not observed in the ATTR_wt_ group. The CVRR_diff rate_ levels in the ATTR_wt_ group were significantly lower than those in the other two groups (ATTR_wt_: −8.77 [−43.8 to 10.9]; LVH: 67.4 [38.7 to 89.4]; MI: 83.7 [60.4 to 142.9]; Figure 2). Based on the ROC analysis the best CVR‐R_diff rate_ cut‐off value with which to identify ATTR_wt_ in HF was 19.7 (AUC: 0.848). Furthermore, at this cut‐off value the sensitivity and specificity for predicting ATTR_wt_ were 97.5% and 80%, respectively. There were no statistically differences in the AUC values between CVR‐R_diff rate_ and troponin I. Analysis of interplay between CVR‐R values and MRI could not be performed due to the small number. There was no significant correlation between CVR‐R and LVMI (data not shown).

**Figure 3:**
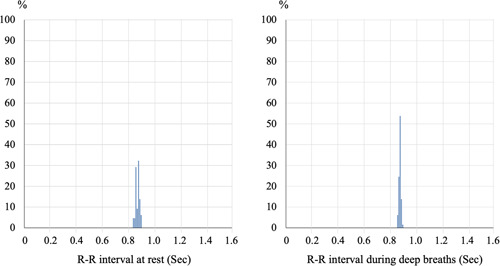
A representative patient with cardiac wild‐type transthyretin amyloidosis (ATTR_wt_). A histogram of RR did not demonstrate the fluctuation in R‐R interval coefficient of variation (CVR‐R) under deep breathing. The CVR‐R_rest and breath_ values were 1.67% and 0.89%. The calculated CVR‐R_diff rate_ was −46.7%.

**Figure 4:**
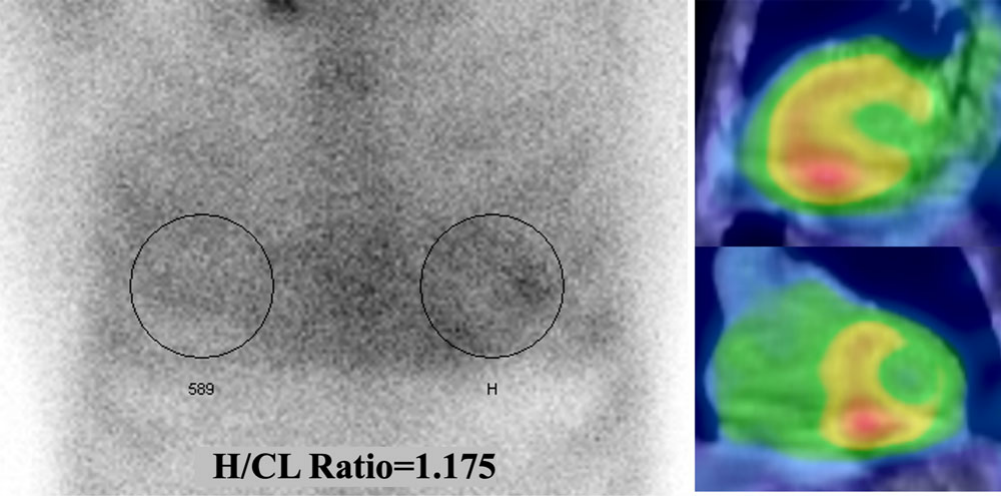
^99m^Technetium‐pyrophosphate scintigraphy showed high cardiac uptake greater than rib uptake (grade 3). The quantitative value of myocardial‐to‐contralateral lung uptake ratio at 3 h was 1.175. H/CL, myocardial‐to‐contralateral lung uptake ratio.

**Figure 5:**
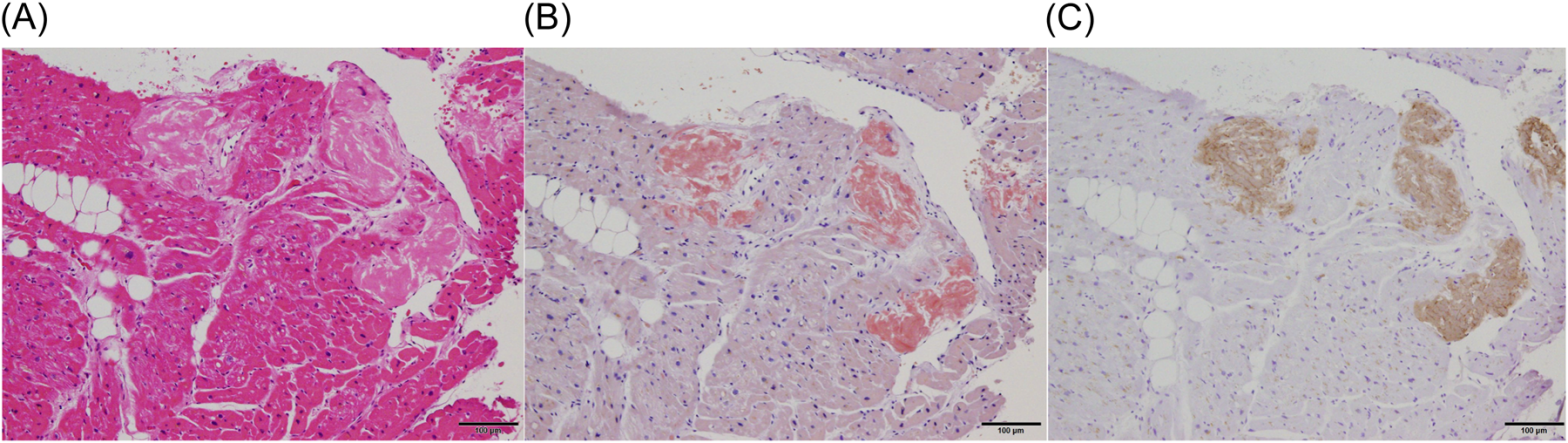
Endomyocardial biopsy. Amyloid deposition was confirmed by endomyocardial biopsy with different staining: hematoxylin and eosin stain (A), congo red stain (B), and immunohistochemical staining for transthyretin (C).

Figures 3, 4, 5 show a representative case of ATTR_wt_. Electrocardiogram findings (e.g., low voltage in the limb leads and a wide QRS) and elevation of high‐sensitive troponin I (162.52 pg/ml) were compatible with ATTR_wt_. Based on echocardiographic data, LVEF, LVMI, E/A, and E wave deceleration time were 37%, 124.5 g/m^2^, 1.53 and 180 ms, respectively; however, apical sparing and RV free wall thickness were not observed. The R‐R histogram showed poor fluctuation of R–R intervals under deep breathing (Figure 3). The CVR‐R_rest_, CVR‐R_breath,_ and CVR‐R_diff rate_ values were 1.67%, 0.89%, and −46.7%, respectively. ^99m^Tc‐PYP scintigraphy showed high cardiac uptake (grade 3). The quantitative value of myocardial‐to‐contralateral lung uptake ratio at 3 h was 1.175 (Figure 4). The diagnosis of ATTR_wt_ was confirmed by endomyocardial biopsy (Figure 5).

## 4 DISCUSSION

The validity of autonomic function test in the pathological assessment of ATTR_v_ has been established.^16^ As for ATTR_wt_, on the other hand, the data is still scarce. To our best knowledge, this study is the first report about the usefulness of deep breath induced CVR‐R fluctuation in the patients with ATTR_wt_. Diagnostic delay in ATTR_wt_ not only precludes adequate therapy, including tafamidis, but also increases healthcare costs.^17^ This method could be one of the clues for early diagnosis of ATTR_wt_.

Measurement of the CVR‐R is a quantitative procedure to evaluate cardiac parasympathetic activation.^18^, ^19^ The CVR‐R is routinely used in Japan to assess the cardiac ANS in patients with diabetes.^20^ The CVR‐R values are associated with glycemic variability,^21^ anemia,^22^ renal function,^23^ and orthostatic hypotension^24^ in patients with diabetes. In addition, the utility of the CVR‐R has been reported in patients with Parkinson's disease,^12^, ^25^ gastrointestinal diseases,^26^ and HF.^27^ The advantages of the CVR‐R are convenience, rapid generation of results, and reproducibility. Moreover, the CVR‐R method can be performed, even in an emergency department.^28^

There are few reports involving CVR‐R values in patients with ATTR_wt_ for several reasons. First, major autonomic disorders, such as orthostatic hypotension, constipation, diarrhea, and urinary tract disorders, are often not apparent in patients with ATTR_wt_. Thus, cardiac autonomic disorders might be overlooked. Second, CVR‐R values are affected by various factors, such as age, gender, and CRP levels.^29^, ^30^ HF severity influence CVR‐R values. Third, ATTR_wt_ is known to be associated with atrial fibrillation and atrioventricular block.^31^ HRV test is not suitable for patients with frequent arrhythmias.^32^ We have ruled out patients with significant arrhythmic disorders; however, even a subtle arrhythmia can influence CVR‐R_rest_ values. For this reason, we noticed a fluctuation of the CVR‐R under deep breathing. In healthy adults, HRV is amplified by deep breathing. HRV amplification is extinguished by atropine, but not beta‐blockers.^33^, ^34^ These results suggested that fluctuation of HRV under deep breathing was mediated by parasympathetic nerves. In some patients with ATTR_wt_, CVR‐R_breath_ values were lower than CVR‐R_rest_ values, and CVR‐R_diff rate_ values presented negative values. This result is opposite to that of LVH and MI groups. Decreased CVR‐R_breath_ values might be caused by the deteriorated fluctuation due to severe impaired autonomic function.

The association CVR‐R values with disease severity was not examined in this study. There have been few reports demonstrating the association between deep breath‐induced CVR‐R fluctuation with disease severity. Miyamoto et al. reported the usefulness of the CVR‐R difference under deep breathing in the clinical assessment of atherosclerosis coexisting in patients with type 2 diabetes mellitus and diabetic neuropathy.^35^ This method has been less used for the assessment of heart diseases because interpretation of the values is difficult for multiple confounding factors. Further research is needed to establish the utility of this method in clinical practice.

This study had several limitations. This was a single center study with a small number of subjects. The small number of eligible patients is a direct result of having many strict inclusion/exclusion criteria. The patient with persistent arrythmias or severe HF (NYHA IV) were excluded due to difficulties in interpreting their data. Our study sample size was too small for multivariate analysis. The combination of elevation of high‐sensitivity cardiac troponin T, left ventricular posterior wall thickness, and wide QRS has been reported to raise the pretest probability of ^99m^Tc‐PYP scintigraphy.^36^ We could not analyze the additional value of CVR‐R to these indexes. Prospective studies with a greater number of patients are needed to validate our results.

The association cardiac amyloid load with CVR‐R values was not evaluated because some patients were diagnosed with endomyocardial biopsy, only. The progression of cardiac amyloid load can affect CVR‐R values as well as echocardiographic findings.^37^ High prevalence of atrial fibrillation with advancing ATTR_wt_ makes it difficult to investigate the association CVR‐R values with the stage of ATTR_wt_.^38^

CVR‐R values can be influenced by etiologies of LVH. HCM has been reported to affect autonomic function.^39^ Cardiac amyloid deposition has been reported to be present in ≤15% of patients with AS.^40^ In this study, not all patients with LVH group underwent endomyocardial biopsy and/or scintigraphy. Underdiagnosis of amyloid deposition may have occurred in the LVH group, although the echocardiographic data showed a difference between the ATTR_wt_ and non‐ATTR_wt_ groups. Genetic testing for ATTR_v_ was not performed for all patients. Finally, late‐onset ATTR_v_ is a possibility that we considered.

## 5 CONCLUSIONS

In this study, decreased CVR‐R levels under deep breathing are highly suggestive of ATTR_wt_. Treatment options for ATTR_wt_ are expanding rapidly. Early ATTR_wt_ diagnosis is necessary for treatment optimization. Measurement of the CVR‐R in HF patients may be a convenient support tool for the detection of ATTR_wt_.

## AUTHOR CONTRIBUTIONS

**Yasuhiro Nagayoshi**: Conceptualization; data curation; formal analysis; investigation; methodology; project administration; writing – original draft; writing – review and editing. **Hiroaki Kawano**: Conceptualization; supervision; writing – original draft; writing – review and editing. **Taiki Nishihara**: Investigation. **Kei Morikawa**: Investigation. **Haruka Nagano**: Investigation. **Shinsuke Hanatani**: Investigation. **Naritsugu Sakaino**: Investigation. **Kenichi Tsujita**: Supervision.

## CONFLICT OF INTEREST

The authors declare no conflict of interest.

## ETHICS STATEMENT

All procedures were performed in accordance with the Declaration of Helsinki and its amendments. The study protocol was approved by the Institutional Review Board of the Amakusa Medical Center (approval no. 20210316‐5).

## TRANSPARENCY STATEMENT

The lead author Yasuhiro Nagayoshi affirms that this manuscript is an honest, accurate, and transparent account of the study being reported; that no important aspects of the study have been omitted; and that any discrepancies from the study as planned (and, if relevant, registered) have been explained.

## Data Availability

Participants of this study did not agree for their data to be shared publicly, so supporting data are not available.
